# Editorial: Physical Activity and Type 1 Diabetes

**DOI:** 10.3389/fendo.2019.00860

**Published:** 2019-12-06

**Authors:** Johan H. Jendle, Michael C. Riddell

**Affiliations:** ^1^Institute of Medical Sciences, Örebro University, Örebro, Sweden; ^2^Diabetes Endocrinology and Metabolism Research Center, Örebro University, Örebro, Sweden; ^3^Muscle Health Research Centre, School of Kinesiology and Health Science, York University, Toronto, ON, Canada; ^4^LMC Diabetes and Endocrinology, Toronto, ON, Canada

**Keywords:** diabetes, physical excercise, CGM (continuous glucose monitoring), hypoglyceamia, treatment—drug

Exercise therapy is a cornerstone for the management of both type 2 (T2D) and type 1 (T1D) diabetes at all ages. Regular physical activity (PA) is associated with many well-established health benefits in individuals with diabetes as well as in individuals without diabetes. PA has been shown to improve cardiovascular fitness, metabolic and bone-health and enhance psychological well-being (1–3) in T2D. Furthermore, regular PA, in combination with other lifestyle changes such as caloric restriction, may prevent or delay T2D development more effectively than pharmacological interventions (4–6).

In T1D, regular PA associates with several positive physical health effects such as improved cardiovascular fitness and blood lipid profile and also enhances psychological well-being (7, 8). However, the beneficial effects on health-related outcomes induced by PA has not been convincingly paralleled with improvements in glycaemic control (7, 9, 10). In addition to ambiguities in the effects that PA has on glucose control, challenges remain as different forms of PA require different approaches regarding management of nutrition and insulin prior to, during and after PA. Adverse reactions to PA such as increased glucose variability, hypo and hyperglycemia are burdens for many patients.

Most adults with diabetes, both T1D and T2D, are recommended to perform at least 150 min of moderate-to-vigorous intensity activity weekly, and the activity should be spread over at least 3 days/week, with no more than 2 consecutive days without activity. Shorter durations (minimum 75 min/week) of vigorous intensity or interval training may be sufficient for complication free and more physically fit individuals (11–13).

What is the best type of exercise? Most likely the exercise that is being performed regularly but there are differences in how the body responds metabolically. It is still unclear as to what form, duration and intensity should be recommended for health, fitness and glycemic control and whether there is a clinically significant benefit for the various clinical outcomes examined. A combination of both aerobic, resistance training and bone strengthening activities is likely to be most effective, but the evidence is not yet convincing for any or all of these types.

New diabetes technologies, including the use of continuous and intermittent flash glucose monitoring, is helpful when it comes to reducing some of the burden around glucose management for sport and exercise. Hybrid closed loop insulin pumps (HCL) and sensor-augmented insulin pumps with automatic basal rate suspension might further facilitate the situation and reduce some of the challenges during and after exercise in T1D (14, 15).

The pharmacological treatments options are now abundant for treatment of T2D and a patient-centered approach should be used to guide the individual choice of glucose lowering agents. If the patient is engaged in sporting activities or has problems with hypoglycemia, for instance during PA, a glucose lowering agent with little risk of inducing hypoglycemia is recommended (16).

This special issue of *physical activity and diabetes* covers a broad field of clinical- and pre-clinical studies to better understand the effects of PA in individuals with diabetes and to better overcome the problems associated with PA in order to increase physical activity adoption.

Moser et al. demonstrate in “Different Heart Rate Patterns During Cardio-Pulmonary Exercise (CPX) Testing in Individuals with Type 1 Diabetes” that there are clear differences in the heart rate response during cardio-pulmonary exercise (CPX) testing in individuals with T1D compared to controls and suggest using other submaximal markers than rated perceived exertion and submaximal heart rate to prescribe exercise intensity in people with T1D.

Yardley et al. provide a review on possible variables for glucose control entitled “Could Age, Sex and Physical Fitness Affect Blood Glucose Responses to Exercise in Type 1 Diabetes?” and conclude that there is currently insufficient information to model a closed-loop system (HCL) that can predict the potential influence of variables accurately and consistently prevent hypoglycaemia.

Matson et al. in “Objective Measurement of Physical Activity in Adults with Newly Diagnosed Type 1 Diabetes and Healthy Individuals” conclude that adults recently diagnosed with T1D do less moderate-to-vigorous-physical-activity (MVPA) than age-matched controls. Health care workers should therefore encourage these people to engage in more PA.

Tagougui et al. discuss in their review “The Benefits and Limits of Technological Advances in Glucose Management Around Physical Activity in Patients Type 1 Diabetes” how new technological advances in insulin delivery devices and glucose monitoring could be used to help glucose management as it relates to physical activity in T1D.

Klaprat et al. describe in “Gaps in Knowledge and the Need for Patient-Partners in Research Related to Physical Activity and Type 1 Diabetes: A Narrative Review” epidemiological evidence on the benefits of PA and T1D and also identify gaps in research aiming to add information to form future guidelines. The authors also provide an overview of patient-oriented research projects co-developed with persons living with T1D.

Litchfield et al. point out, in their paper entitled “Patient and Healthcare Professionals Perspectives on the Delivery of Exercise Education for Patients with Type 1 Diabetes,” that any education package developed to support exercise in patients with T1D should be offered at a time following diagnosis in accordance with patients' preferences and priorities, and also provide information on how to manage regular and irregular bouts of exercise.

Chetty et al. provide, in their review “Exercise Management for Young People with Type 1 Diabetes: A Structured Approach to the Exercise Consultation,” a structured approach on how to preform exercise consultations for children/adolescents based on a framework of questions that assist the health care professionals in formulating person-specific exercise management plans for young people with T1D.

McCarthy et al. discuss in their review “Resistance Isn't Futile: The Physiological Basis of the Health Effects of Resistance Exercise in Individuals With Type 1 Diabetes” the health benefits and challenges of resistance exercise in individuals with T1D.

Finally, Mattsson et al. present an original study entitled “Carbohydrate Loading Followed by High Carbohydrate Intake During Prolonged Physical Exercise and its Impact on Glucose Control in Individuals with Diabetes Type 1—an Exploratory Study” that shows that high volume intermittent CHO feeding during prolonged PA, combined with proactive use of continuous glucose monitoring (rtCGM), is associated low glucose variability and excellent glycemic control during extreme prolonged exercise in athletes with T1D.

## Author Contributions

The editorial article has been written by JJ. MR has reviewed and approved the manuscript and co-edited the special issue on PA and diabetes.

### Conflict of Interest

The authors declare that the research was conducted in the absence of any commercial or financial relationships that could be construed as a potential conflict of interest.
